# Notes and News

**Published:** 1906-04-07

**Authors:** 


### Notes and News.

An anonymous donor has given £10,000 to King Edward's Hospital Fund for London. The amount is to be applied to the capital fund.

The children of the late Mr. T. Dowsett have given £1,000 towards the cost of erecting a children's ward at the Victoria Hospital, Southend-on-Sea.

A new Isolation Hospital has been built at Stanfield, for the borough of Burslem. It is not yet furnished, but on the occasion of the recent formal opening a block was fully equipped to show visitors what the general arrangement of the hospital will be when completed.

The London Brighton and South Coast Railway Company gives notice that it will issue return tickets from London to Irun to members of the International Congress of Medicine and their wives and children. The tickets are available for sixty days *via* Dieppe-Paris and Bordeaux at fares £5 15s. 5d. first class and £4 1s. second class. They can be obtained at the Victoria and London Bridge Stations of the L.B. & S.C.R., the only formality necessary being the presentation of the *carte d'identité*.

Samples of milk supplied to the City of London have recently been the subject of a bacteriological investigation by Dr. Klein. Twenty-five samples were examined, and of these twenty were satisfactory, two were tuberculous, and three were regarded as unsatisfactory. The medical officer, in his report, says the results are further proof of the utterly reckless and careless way in which most of the milk trade is carried on, and he further expresses the opinion that the Legislature should devise some practical means of dealing with "this grave source of danger."

The South Eastern and Chatham Railway Co. has arranged conjointly with the French railways to issue return tickets from London to Irun at the greatly reduced rates of £6 13s. first class, and £4 12s. second class, for members who propose to travel overland to Lisbon to attend the fifteenth International Medical Congress. The tickets will be available via Dover and Calais or by Folkestone and Boulogne, at the option of the passenger; but members are advised very strongly to take the 10 a.m. service from Victoria via Folkestone and Boulogne, as that service is the least crowded, and reaches Paris at 6.4 p.m., in ample time to dine before continuing the journey by the 8.7 p.m. express from the Quai d'Orsay station. This train reaches Irun at 8.50 a.m. on the following morning. The tickets are available for 45 days, and will also be issued to the wives and children of members, but not to other relatives. They can only be obtained at the Victoria S.E. and C.R. on production of the membership certificates from April 1 onwards.

